# Prostaglandin E-Major Urinary Metabolite (PGE-MUM) as a Tumor Marker for Lung Adenocarcinoma

**DOI:** 10.3390/cancers11060768

**Published:** 2019-06-03

**Authors:** Hironori Kawamoto, Hiromichi Hara, Jun Araya, Akihiro Ichikawa, Yu Fujita, Hirofumi Utsumi, Mitsuo Hashimoto, Hiroshi Wakui, Shunsuke Minagawa, Takanori Numata, Seiji Arihiro, Tomokazu Matsuura, Mutsunori Fujiwara, Satoru Ito, Kazuyoshi Kuwano

**Affiliations:** 1 Division of Respiratory diseases, Department of Internal Medicine, School of Medicine, Jikei University, Tokyo 105-8471, Japan; h5amm1212@yahoo.co.jp (H.K.); araya@jikei.ac.jp (J.A.); a-ichikawa@jikei.ac.jp (A.I.); yuugot@gmail.com (Y.F.); hiro173@jikei.ac.jp (H.U.); mitsuoha-georgetown@live.jp (M.H.); hwakui0304@gmail.com (H.W.); shunsuke@jikei.ac.jp (S.M.); t-numata@xa2.so-net.ne.jp (T.N.); kkuwano@jikei.ac.jp (K.K.); 2 Division of Gastroenterology and Hepatology, Department of Internal Medicine, School of Medicine, Jikei University, Tokyo 105-8471, Japan; sarihiro@gmail.com; 3 Department of Laboratory Medicine, School of Medicine, Jikei University, Tokyo 105-8471, Japan; matsuuratomo@gmail.com; 4 Department of Clinical Pathology, Japanese Red Cross Medical Center, Tokyo 150-8935, Japan; m-fujiwara@mbg.ocn.ne.jp; 5 IDAC Theranostics, Inc.; Tokyo 113-0033, Japan; sr-ito@idac-thera.com

**Keywords:** PGE-MUM, lung adenocarcinoma, biomarker

## Abstract

*Background*: Prostaglandin E2 (PGE2) is metabolized to prostaglandin E-major urinary metabolite (PGE-MUM). Enhanced cyclooxygenase-2 (COX-2) expression demonstrated in lung adenocarcinoma indicates increased PGE-MUM levels in patients with lung adenocarcinoma. *Objectives*: We aimed to elucidate the clinical usefulness of measuring PGE-MUM as an indicator of tumor burden in patients with lung adenocarcinoma. *Methods:* PGE-MUM was measured by a radioimmunoassay in control healthy volunteers (*n* = 124) and patients with lung adenocarcinoma (*n* = 54). Associations between PGE-MUM levels and clinical characteristics of the patients (including lung cancer stage and TNM factors (T: Tumor, N: Node, M: Metastasis) were examined. *Results:* PGE-MUM levels were significantly elevated in patients with lung adenocarcinoma. A PGE-MUM level of 14.9 μg/g∙Cr showed 70.4% sensitivity and 67.7% specificity for the diagnosis of lung adenocarcinoma. PGE-MUM levels tended to be positively correlated with cancer progression as determined by the TNM staging system. Advanced stage (stage III, stage IV, and recurrence) was significantly associated with high PGE-MUM levels by logistic regression analysis. No apparent correlation was demonstrated between PGE-MUM and carcinoma embryonic antigen (CEA) levels. *Conclusions*: PGE-MUM can be a promising biomarker reflecting the systemic tumor burden of lung adenocarcinoma.

## 1. Introduction

Lung cancer is one of the leading causes of death worldwide, and cigarette smoking is the main risk factor for lung cancer [1]. Non-small-cell lung cancer (NSCLC) is the most common type of lung cancer and comprises approximately 80% of lung cancer cases [1]. Recent advances in NSCLC treatment modalities, including immunotherapy, improve survival overtime [2], resulting in increased opportunities for selection of treatment modalities. To precisely evaluate treatment efficacy, tumor markers reflecting tumor burden are indispensable during lung cancer therapy.

Prostaglandin E2 (PGE2) is synthesized by cyclooxygenase (COX) and metabolized to stable prostaglandin E-major urinary metabolite 7alpha-hydroxy-5,11-diketotetranor-prosta-1,16-dioic acid (PGE-MUM) [3]. COX-2 is involved in lung tumorigenesis in mouse models, and PGE2 has been shown to promote proliferation and invasion of cancer cells [4,5,6]; hence, the COX-2/PGE2 pathway can be of great importance in cancer patients who have elevated COX-2 levels. To evaluate the COX-2/PGE2 pathway, a reproducible method for measuring stable PGE-MUM using a simple radioimmunoassay kit was recently developed. The clinical usefulness of PGE-MUM measured by this kit as a biomarker showing disease activity has been demonstrated in inflammatory bowel disease [7] and chronic fibrotic lung disease [8].

Several lines of evidence have suggested that COX-2 expression is increased in NSCLC, especially adenocarcinoma [9,10,11], and that higher COX-2 expression levels are associated with worse outcomes [10,12]. Accordingly, increased COX-2 expression may be linked to enhanced PGE-MUM, reflecting tumor burden in patients with lung adenocarcinoma. In this study, we explored the clinical usefulness of measuring PGE-MUM in patients with lung adenocarcinoma.

## 2. Materials and Methods

### 2.1. Samples and Data Collection

Fifty-four patients diagnosed with lung adenocarcinoma (stage I: 26, stage II: 3, stage III: 7, stage IV: 14, recurrence after surgery: 4) participated in this study at the Jikei University Hospital, Tokyo, Japan, between May 2013 and August 2018. Patients with complications such as active inflammatory disease or uncontrolled cancer of other organs were excluded. The medical records of the patients were reviewed. Healthy volunteers (*n* = 124) in the Japanese Red Cross Medical Center were enrolled as normal controls. The ethics committee approval was obtained in Jikei University and the Japanese Red Cross Medical Center (24-323 7089). Written informed consent was obtained from all participants. Spot urine samples were centrifuged at 1000× *g* for 10 min, and the supernatants were stored at −20 °C until analysis.

### 2.2. Measurement of PGE-MUM

PGE-MUM levels were measured by a radioimmunoassay kit (Institute of Isotopes Co., Ltd, Budapest, Hungary). Briefly, after alkaline treatment followed by neutralization of the urine samples, synthesized bicyclic PGE-MUM was measured by a competitive assay. PGE-MUM levels were normalized to the concentration of urinary creatinine (expressed as μg/g∙Cr). PGE-MUM levels pre- and post-lung resection were measured in cases with surgical treatment (post-lung resection samples were obtained three months after surgery). PGE-MUM levels pre- and post-systemic therapies were measured in cases with systemic therapy (samples of post-systemic therapy were obtained two months after the initiation of the systemic therapy).

### 2.3. Statistical Analysis

Statistical procedures except, ROC curve analysis, were performed using StatView version 5 (SAS Institute Inc., Cary, NC, USA) and EXEL 2013. ROC curve analysis was performed with EZR (Saitama Medical Center, Jichi Medical University, Saitama, Japan).

Student’s *t* test and analysis of variance followed by Bonferroni’s post-hoc comparison tests were performed to compare PGE-MUM levels among groups.

We used multiple regression analysis to evaluate the effect of patient characteristics on PGE-MUM levels in lung cancer patients. A correlation between patient characteristics and high PGE-MUM level was examined using a Fisher’s exact test, Student’s *t* test, or chi-squared test (univariate model). Then, a logistic regression analysis was performed to evaluate factors associated with high PGE-MUM (multivariate model), including age, sex (male), and other variables that achieved *p* < 0.20 in the univariate models; *p*-values of less than 0.05 were considered statistically significant.

## 3. Results

### 3.1. Clinical Characteristics of the Patients

PGE-MUM levels in 124 healthy volunteers and 54 patients with lung adenocarcinoma were measured. Age (mean ± SD), sex (male %), BMI (Body mass index), and smoking history in each group are shown in Table 1. The participants in the control group were significantly younger than in the lung adenocarcinoma group (control; 44.2 ± 12.9 years, lung adenocarcinoma; 66.6 ± 10.0 years). There was no difference in the sex ratio between the control group (male: *n* = 52, female: *n* = 72) and the lung adenocarcinoma group (male: *n* = 23, female: *n* = 31). BMI was higher in the lung adenocarcinoma group (control; 22.3 ± 3.1, lung adenocarcinoma; 21.0 ± 3.2 years); 55.6% of lung adenocarcinoma group had smoking history.

### 3.2. PGE-MUM Levels in Lung Adenocarcinoma

**Figure 1:**
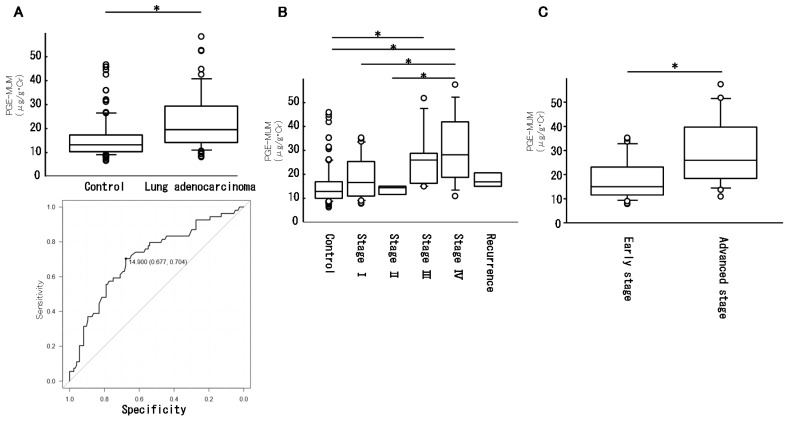
Prostaglandin E-major urinary metabolite (PGE-MUM) levels in lung adenocarcinoma. PGE-MUM levels in control participants and patients with lung adenocarcinoma are shown ((**A**); top panel). The bottom panel in A is the result of ROC curve analysis. Patients with lung adenocarcinoma were classified into five categories (stage I, II, III, IV, recurrence), and the PGE-MUM levels in each group are shown in (**B**). A comparison of PGE-MUM levels in patients with advanced-stage disease (III, IV, and recurrence) and early-stage disease (I and II) is shown in (**C**). (* *p* < 0.05).

PGE-MUM levels were significantly increased in patients with lung adenocarcinoma (control: 15.4 ± 8.3 μg/g∙Cr, lung adenocarcinoma: 22.4 ± 11.9 μg/g∙Cr, *p* < 0.0001, Figure 1A top). On the basis of the ROC curve analysis, a PGE-MUM cut-off level of 14.9 showed 70.4% sensitivity and 67.7% specificity, which might be the optimal cut-off point for lung adenocarcinoma diagnosis (Figure 1A bottom). The lung adenocarcinoma group was divided into five categories (stage I–IV and recurrence). The PGE-MUM levels in each category were as follows: stage I: 18.6 ± 8.8, stage II: 13.3±2.5, stage III: 26.2 ± 12.7, stage IV: 30.8 ± 14.4, and recurrence: 17.8 ± 3.6 (Figure 1B). The PGE-MUM levels in stage IV patients were significantly higher than in the control group, stage I, and stage II patients. The PGE-MUM levels in stage III patients were significantly higher than in control participants. The PGE-MUM levels in patients with advanced stage disease (stage III, stage IV, recurrence) were significantly higher than in patients with early-stage disease (stage I and stage II) (early stage: 18.1 ± 10, advanced stage: 27.4 ± 13.3, *p* = 0.003, Figure 1C). When a PGE-MUM level of 14.9 was used as a cut-off level, the positive rate in each stage was 57.7% (stage I, *n*  = 26), 66.7% (stage II, *n*  = 3), 85.7% (stage III *n*  = 7), 85.7% (stage IV, *n*  = 15), and 75.0% (relapsed, *n*  = 4). The negative rate of the control group was 72.6%.

### 3.3. Association of PGE-MUM Levels with TNM Factors

**Figure 2:**
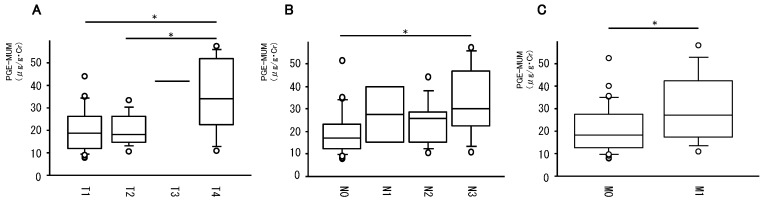
Association of PGE-MUM levels with TNM factors. The association of PGE-MUM levels with T-factor (**A**), N-factor (**B**), and M-factor (**C**) is shown. (* *p* < 0.05)

We investigated the association of PGE-MUM levels with each TNM factor (Figure 2). PGE-MUM levels associated with T4 were significantly higher than those associated with T1 and T2 (Figure 2A). PGE-MUM levels associated with T3 tended to be higher than those associated with T1 and T2. PGE-MUM levels associated with N3 were significantly higher than that associated with N0 (Figure 2B). PGE-MUM levels associated with N1 and N2 tended to be higher than those associated with N0. PGE-MUM levels associated with M1 were significantly higher than those associated with M0 (Figure 2C). Taken together, elevated PGE-MUM levels were associated with progression of each TNM factor.

### 3.4. Influence of Lung Adenocarcinoma Treatments on PGE-MUM Levels

**Figure 3:**
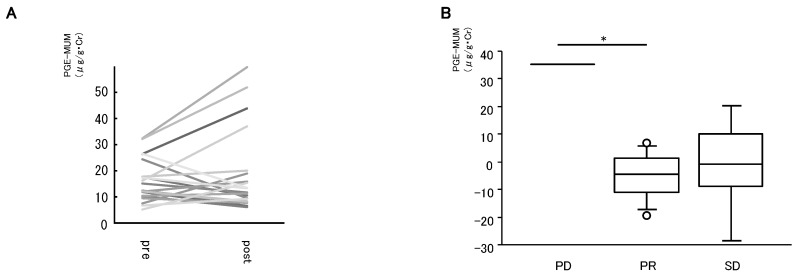
Association of PGE-MUM levels with treatment. Changes in PGE-MUM levels after lung resection are shown in (**A**) (*n* = 21). The patients treated with systemic therapies were classified into three groups according to their response to treatment (progressive disease, PD *n* = 1, partial response, PR *n* = 8, stable disease, SD *n* = 5), and the changes in PGE-MUM levels after systemic therapies in each group are shown in (**B**). (* *p* < 0.05).

Next, the influence of lung adenocarcinoma treatments on PGE-MUM levels was examined (Figure 3). PGE-MUM levels pre- and post-lung resection were compared. Changes in PGE-MUM levels after lung resection are shown in Figure 3A (*n* = 21). In several cases, PGE-MUM increased without tumor progression, partly because of persistent perioperative inflammation; however, there was no significant change in PGE-MUM levels after lung resection.

In advanced cases, systemic therapies including chemotherapy, chemoradiotherapy, and molecular targeted therapy were performed (chemotherapy: *n* = 7, chemoradiotherapy: *n* = 4, EGFRTKI: *n* = 7, ALK inhibitor: *n* = 1, immunotherapy: *n* = 2, BSC: *n* = 1). In 14 cases, PGE-MUM levels pre- and post-systemic therapies were evaluated (chemotherapy: *n* = 6, chemoradiotherapy: *n* = 1, EGFRTKI: *n* = 5, ALK inhibitor: *n* = 1, immunotherapy: *n* = 1). Treatment response was evaluated after 2 cycles of standard chemotherapy in patients treated with chemotherapy. The patients were classified into three groups according to their response to treatment (progressive disease, PD *n* = 1; partial response, PR *n* = 8; stable disease, SD *n* = 5). The changes in PGE-MUM levels after systemic therapies in each group are shown in Figure 3B. The changes in PGE-MUM levels were +35.3 in PD, −5.1 ± 8.6 in PR, and −1.0 ± 17.8 in SD, respectively. The changes in PD were significantly higher than those in PR.

### 3.5. Multivariate Analysis of Factors Associated with High PGE-MUM

To determine factors associated with high PGE-MUM (≥ 22.0), we performed univariate analysis (Table 2) and multivariate analysis (Table 3) in patients with lung adenocarcinoma. In univariate analysis, the effects of clinical characteristics (age, sex, BMI, smoking history, advanced stage, and use of a COX-2 inhibitor) on PGE-MUM levels were examined. Advanced stage was significantly associated with high PGE-MUM (*p* = 0.02). Patients in the high-PGE-MUM group tended to be older, and the percentage of male sex and smoker in the high-PGE-MUM group tended to be higher, although the differences were not significant. There was no difference in BMI between the groups. We could not detect any effects of the COX-2 inhibitor on PGE-MUM levels, since the COX-2 inhibitor was used in only one patient in the high-PGE-MUM group. 

Next, a multivariate analysis was performed to evaluate factors associated with high PGE-MUM, including age, sex (male), and other variables that achieved *p* < 0.20 in the univariate models. Advanced stage was significantly associated with high PGE-MUM (Table 3).

### 3.6. Correlation between PGE-MUM and Carcinoma Embryonic Antigen (CEA)

**Figure 4:**
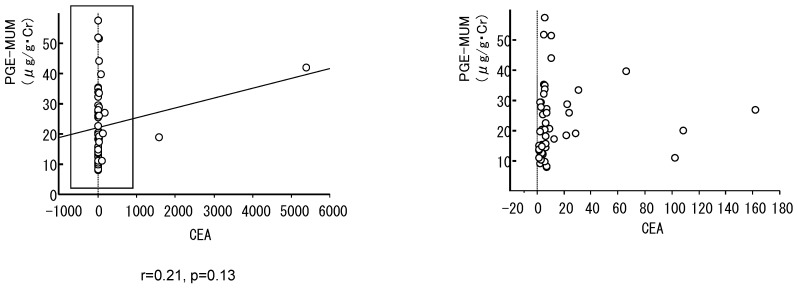
Association of PGE-MUM levels with carcinoma embryonic antigen (CEA). A scatter plot of PGE-MUM and CEA (**left**: whole data, **right**: magnification of the box in the left panel).

CEA is a representative biomarker of lung adenocarcinoma. Thus, we examined the correlation between PGE-MUM and CEA. Figure 4 is a scatter plot of PGE-MUM and CEA levels. PGE-MUM levels did not correlate with CEA (*r* = 0.21, *p* = 0.13), suggesting that PGE-MUM is an independent biomarker for lung adenocarcinoma.

## 4. Discussion

In the present study, we showed significantly increased PGE-MUM levels in patients with lung adenocarcinoma. PGE-MUM levels tended to be positively correlated with cancer progression on the basis of the TNM staging system. Advanced disease stage (stage III, stage IV, recurrence) was significantly associated with high PGE-MUM, suggesting that PGE-MUM can be a promising tumor marker of lung adenocarcinoma reflecting systemic tumor burden. 

PGE-MUM is a stable end product of PGE2 and reflects the amount of systemic PGE2 production. However, PGE-MUM can also indicate locally increased PGE2 production in disease sites in patients without other complicating disorders [7,8]. It is likely that elevated PGE-MUM levels reflect PGE2 production from lung adenocarcinoma because patients were not affected by complications such as active inflammatory disease and cancer of other organs in the present study. Murphey L.J. et al. and Kozak K.R. et al. demonstrated that PGE-MUM levels were increased in cases with unresectable NSCLC [13,14]. However, these studies included a small number of NSCLC patients (19 patients and 29 patients, respectively), and the association with clinical characteristics was not precisely evaluated. Recently, several studies were performed to elucidate the association of PGE-MUM levels with the effect of COX-2 inhibitors [15,16,17,18,19,20,21,22]. Patients in advanced stages were included in these studies; however, the association of PGE-MUM levels with cancer progression was not evaluated. More recently, Shimizu K et al. investigated the association of PGE-MUM with clinical characteristics including cancer stage; however, these authors could not show any association between PGE-MUM levels and clinical characteristics, possibly because of the small sample size (total of 21 patients), tumor heterogeneity (adenocarcinoma: 18, squamous cell carcinoma: 3), and low COX-2 expression in the studied group (high expression: 7, low expression: 14) [23]. To elucidate the clinical usefulness of PGE-MUM as a biomarker reflecting tumor burden, we focused on lung adenocarcinoma, known to express high COX-2 levels [6], and 54 patients were enrolled. In line with a previous report, PGE-MUM levels were significantly elevated in patients with lung adenocarcinoma; we suggest that 14.9 μg/g∙Cr might be the optimal cut-off point with high sensitivity and specificity. PGE-MUM levels tended to be positively correlated with cancer progression, and significant PGE-MUM elevation was demonstrated in patients with advanced-stage disease (III, IV, recurrence). We hypothesize that the total amount of cancer cells that express COX-2 might be large in advanced-stage tumors. Several lines of evidence suggest that age, sex, smoking history, and use of COX-2 inhibitors affect PGE-MUM levels. Advanced stage was more closely associated with high PGE-MUM levels than these clinical factors in this study, suggesting that PGE-MUM levels can directly reflect tumor burden and be considered as a biomarker for lung adenocarcinoma.

PGE-MUM is noninvasively measured using urine. Hence, we hypothesize that PGE-MUM can be optimal for the consecutive assessment of tumor burden of lung adenocarcinoma, especially in the setting of anti-cancer treatment. However, we could not clearly demonstrate the positive correlation between the effect of treatments and PGE-MUM levels in this study. There are several potential reasons for this. First, this can be attributed to the small sample size and short observation period in the present study. Second, tumor burden could be small in the operable cases. In addition, persistent perioperative inflammation overcame the effect of tumor resection. Third, although only one case was included, the PD case showed apparently increased PGE-MUM levels, suggesting that a certain amount of alteration in tumor burden is necessary to detect PGE-MUM changes. Unfortunately, a CR (complete response) case with more drastic alteration of tumor burden was not included in the present study. Accordingly, to elucidate the association between PGE-MUM levels and treatment response, larger studies with longer observation periods should be performed. 

Although we examined PGE-MUM to evaluate the COX-2/PGE2 pathway, immunohistochemical evaluation of COX-2 can be an alternative method to analyze this pathway. Actually, previous studies showed that elevated COX-2 expression levels were associated with worse outcomes in NSCLC cases [10,12,24,25]. However, the usefulness of immunohistochemical evaluation of COX-2 as a biomarker can be limited because of the heterogeneity of COX-2 expression within a sample and the difficulty in repeated evaluations. Generally, tissue samples are obtained only at initial diagnosis. A recent phase III study demonstrated that COX-2 expression levels were not associated with prognosis and described the limited usefulness of immunohistochemical evaluation of COX-2 as a biomarker in NSCLC cases [15].

Carcinoma embryonic antigen (CEA) is one of the currently available biomarkers for lung adenocarcinoma. It has been reported that the sensitivity and specificity of CEA are 69% and 68%, respectively [26]. Similar levels of specificity and sensitivity were achieved for PGE-MUM. Intriguingly, there was no positive correlation between PGE-MUM levels and CEA (*r* = 0.21, *p* = 0.13), suggesting that PGE-MUM and CEA can be mutually exclusive biomarkers for lung adenocarcinoma. Activation of the COX-2/PGE2 pathway and enhancement of CEA production in cancer cells might be controlled by different signals. In addition, PGE-MUM and CEA levels in human samples are affected by different clinical factors. For example, CEA levels are elevated in type 2 diabetes [27], while PGE-MUM levels are significantly higher in males than in females [28].

This study has several limitations. First, this study was retrospectively designed and performed in a single center. Second, the sample size was small with a short observation period. Since available data in control group were limited and sample size was small, we did not match control group and case group. Hence, further studies are needed to determine accurate sensitivity and specificity of PGE-MUM in lung adenocarcinoma. However, diagnosis of lung cancer and male sex were associated with high PGE-MUM levels evaluated by multiple regression analysis (data not shown), suggesting PGE-MUM can be a biomarker for lung adenocarcinoma diagnosis. Third, we excluded patients with complications such as underlying inflammatory disorders and cancers of other organs. Persistent perioperative inflammation hampered the usefulness of this marker in operable cases. Thus, the utility of PGE-MUM as a biomarker might be limited to selected cases without complicating disorders affecting PGE-MUM levels.

## 5. Conclusions

We consider PGE-MUM to be a promising biomarker reflecting the tumor burden of lung adenocarcinoma, especially in cases without complicating disorders.

## Figures and Tables

**Table 1:** Characteristics of lung adenocarcinoma patients and controls. 124 healthy volunteers and 54 patients with lung adenocarcinoma were enrolled. Age, sex (male %), BMI, and smoking history in each group are shown.

	Lung Adenocarcinon (*n* = 54)	Control (*n* = 124)	*p* Value
Age (year)	66.6 ± 10.0	44.2 ± 12.9	<0.001
Male (%)	23 (42.6)	52 (41.9)	>0.99
BMI	22.3 ± 3.1	21.0 ± 3.2	0.017
Smoking History (%)	55.6	N.A.	N.A.
stage			
I	26		
II	3		
III	7		
IV	14		
rec	4		

N.A.: Not available, BMI: body mass index.

**Table 2:** Univariate analysis of clinical factors association of with high PGE-MUM. The effects of clinical characteristics (age, sex, BMI, smoking history, advanced stage, and use of a COX-2 inhibitor) on PGE-MUM levels were examined.

	Low PGE-MUM (*n* = 33)	High PGE-MUM (*n* = 21)	*p* Value
Age (year)	65.2 ± 10.1	68.8 ± 9.7	0.21
Male (%)	12 (36.3)	11 (52.3)	0.25
BMI	22.3 ± 3.2	22.3 ± 2.9	0.98
Smoking History (%)	15 (45.4)	15 (71.4)	0.06
Advanced stage (%)	11 (33.3)	14 (66.7)	0.02
Use of COX-2 inhibitor (%)	0 (0)	1 (0.05)	0.39

**Table 3:** Multivariate analysis of clinical factors association of with high PGE-MUM. The effects of age, sex, smoking history, and advanced stage on PGE-MUM levels were examined.

	Odds	95% CI	*p*-Value
Age	1.076	1.000–1.157	0.051
Sex (male)	2.211	0.495–9.873	0.299
Smoking history	2.8	0.593–13.225	0.194
Advanced stage	4.99	1.306–19.064	0.0187
